# Internal Strangulation of an Intestine

**Published:** 1847-10

**Authors:** 


*Internal Strangulation of an Intestine.*—The following case recorded by Dr. Biaggini of Pestoic, possesses many points of interest, but chiefly from the cause to which it is attributed:—A boy, aged 15, was witnessing some public ceremony in the midst of a great concourse of people; he felt a desire to go to stool, but was constrained to resist from the difficulty of getting out of the crowd, or of finding a convenient place to ease himself. After enduring this for some ime, he felt as if he was exposed to a sharp cold wind, which he attributed to his being thinly clad: in this state he remained for five hours, and at last got an opportunity of retiring; but on trying at stool, he found to his surprise he was totally unable to pass anything from his bowels. He was taken into a house, where glysters were thrown up, and the abdomen was fomented. For two days he remained without any alleviation, although the enemata brought away a trifling quantity of soft fæces. The belly now became swollen and tympanic,—there was constant vomiting, and a smart fever set in; in short the patient presented all the symptoms of internal strangulation of the bowels. The whole belly was sonorous on percussion above the umbilicus, but gave a dull sound below this point. Notwithstanding a variety of treatment, the distress continued until the twelfth day, when he expired.

*Dissection.*—The peritoneum on both its visceral and parietal surfaces showed traces of very high inflammation, and there was a quantity of curdy serum in its cavity; the intestines adhered here and there by soft and recent lymph. These adhesions were carefully broken down, and it was seen that the distension of the bowel, which existed in the upper part of the canal, ceased abruptly at the lower extremity of the ileum; at this point there was a firm tumour formed by a twisting of a knuckle of gut, covered throughout by recent bands. Below this tumour the large intestine was so contracted as not to be thicker than a ribon, or even a cat's bowel. In detaching the bands with care, that surrounded the tumour, it was found to be formed in the following manner—the mensetery was pierced by an aperture above the cœcum; it was in this hole that the knuckle of intestine was held and strangulated, and through which a portion, about a palm in length, had passed; it was twisted upon itself and was firmly fixed in its situation by the bands above mentioned. The mucous membrane of the intestine was injected, except where it was constricted by the part in which it was held.—*Ibid, from Arch. Gen.*

